# Influence of climate and heatwaves on dengue transmission in Sao Paulo and Natal, Brazil

**DOI:** 10.1371/journal.pone.0334838

**Published:** 2025-10-24

**Authors:** Camila Lorenz, Rita Yuri Ynoue, Adriana Gioda, Thiago Nogueira

**Affiliations:** 1 Faculdade de Saúde Pública da Universidade de São Paulo, São Paulo/SP, Brasil; 2 Instituto Butantan, São Paulo/SP, Brasil; 3 Instituto de Astronomia, Geofísica e Ciências Atmosféricas da Universidade de São Paulo, São Paulo/SP, Brasil; 4 Pontifícia Universidade Católica do Rio de Janeiro, Rio de Janeiro/RJ, Brasil; Universidad Cooperativa de Colombia, COLOMBIA

## Abstract

Dengue fever, a mosquito-borne viral disease, poses a significant public health challenge whose transmission dynamics are highly sensitive to climatic conditions. However, the effects of extreme weather events like heatwaves remain poorly understood. This study investigated the influence of climatic factors and heatwaves on dengue incidence in two key Brazilian hotspots: the subtropical megacity of São Paulo (Sao Paulo State) and the tropical coastal city of Natal (Rio Grande do Norte State). We analyzed weekly confirmed dengue cases and meteorological data (temperature, precipitation, heatwaves) from 2014 to 2023. Distributed lag non-linear models and negative binomial regression were used to assess the complex, delayed associations between meteorological variables and dengue infections. Over the study period, 149,468 dengue cases were reported in São Paulo and 80,999 in Natal. Transmission patterns differed significantly, with Natal exhibiting more regular epidemic cycles. Our models revealed that higher minimum temperatures were associated with increased dengue risk in both cities. Conversely, and perhaps counter-intuitively, higher maximum temperatures and total precipitation showed negative associations with dengue cases. The impact of heatwaves was strikingly different between the locations. In São Paulo, the occurrence of a heatwave was associated with a 70% reduction in dengue risk in subsequent weeks (Relative Risk [RR]: 0.30, 95% Confidence Interval [CI]: 0.18–0.49). In contrast, no statistically significant association between heatwaves and dengue was observed in Natal. Our findings demonstrate that the relationship between extreme heat and dengue transmission is not uniform and can be inhibitory, challenging the assumption that warming consistently favors vector proliferation. These location-specific insights are critical for developing more accurate, tailored public health early-warning systems and caution against one-size-fits-all climate adaptation strategies for vector-borne diseases.

## Introduction

Dengue, the most prevalent virus transmitted by mosquitoes and a major global health issue, impacts more than 126 nations and incurs yearly expenses amounting to billions of US dollars [1–3]. While vector control remains the most practical strategy for limiting dengue transmission, it has not been sufficient to stop the escalating frequency of arbovirus epidemics or the widening geographic range of endemic transmission [3]. This persistent expansion is largely fueled by well-documented factors, including increased air travel, intensified international trade, and growing urban overcrowding [4,5]. Growing attention from researchers and policymakers is now focused on the role of weather conditions in dengue outbreaks, particularly in the context of climate change [3,6,7].

Growing data from epidemiological and experimental studies suggests a relationship between ambient temperature and dengue occurrence [8]. The correlation between temperature and dengue occurrence exhibits a bell-curve pattern, where the peak risk emerges at an ideal temperature, commonly known as the inflection threshold [9,10]. Although dengue occurrence demonstrates a distinct seasonal trend, prior research examining the short-term influence of temperature on dengue cases has generally encompassed all recorded instances within the study timeframe or peak transmission phases [9,10]. While the relationship between maximum temperatures and dengue prevalence in tropical regions has been widely explored [10–14], investigations into the effects of heatwaves remain scarce. Heatwaves, described as periods of extreme heat exceeding a specific threshold, may influence dengue distribution differently from isolated high-temperature events. For example, extreme heat during heatwaves has been shown to inhibit mosquito abundance [15]. Additionally, high temperatures can suppress viral replication and mosquito activity while prompting human behavioral changes that reduce transmission risks – a pattern supported by a study [9] that demonstrated the inhibitive effect of heatwaves on mosquito populations. In contrast, research by Cheng et al. [3] conducted in subtropical Hanoi, Vietnam, indicated that heatwaves may contribute to more severe dengue outbreaks at a later time. This evidence indicates a potential association between heatwaves and dengue occurrence. As climate change accelerates in the 21st century, heatwaves are projected to become more frequent, intense, and prolonged [16], highlighting the urgent need to understand their impact on dengue outbreaks.

Dengue is a growing global health threat, with cases rising by 85.5% from 1990 to 2019 – up from 30.7 million to 56.9 million – and deaths increasing from 28,151–36,055 in the same period [17]. The Americas recorded a historic peak in 2023, with 4.5 million cases and over 2,300 deaths [18]. In Brazil, where dengue re-emerged in 1986 [19], transmission has intensified in recent years, fueled by the widespread presence of mosquito vectors and the co-circulation of all four virus serotypes [20]. While temperature is a well-established factor in dengue transmission, the role of extreme heat events – particularly heatwaves – remains poorly understood. Unlike general warming trends, heatwaves represent short-term, intense spikes in temperature that may uniquely influence mosquito behavior, virus replication, and human vulnerability. This study investigates the short-term associations between heatwaves and dengue incidence in Brazil, focusing on two urban centers with distinct climatic and socioeconomic profiles.

We analyze data from São Paulo and Natal, two dengue-endemic cities in Brazil experiencing rising case numbers, to: (1) evaluate the impact of temperature, precipitation, and heatwaves on dengue incidence; (2) compare patterns across contrasting regional contexts; and (3) evaluate whether heatwaves could serve as early indicators of impending outbreaks. By examining heatwaves as discrete climatic stressors, this study contributes new evidence on how climate extremes – beyond long-term averages – may shape arbovirus dynamics in urban environments.

## Materials and methods

### Study area and data collection

**Fig 1:**
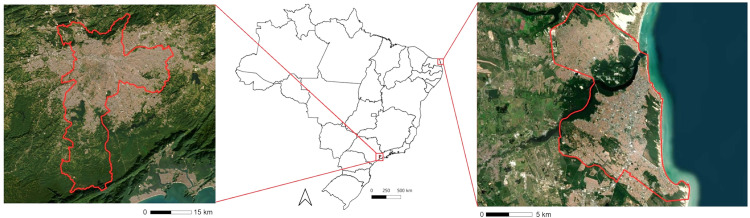
Study area in Brazil. Left: Sao Paulo city, capital of Sao Paulo State. Right: Natal city, capital of Rio Grande do Norte State. Source of map base layers (public domain): IBGE: https://www.ibge.gov.br/geociencias/cartas-e-mapas/mapas-estaduais.html and USGS EROS (Earth Resources Observatory and Science Center): https://earthexplorer.usgs.gov/ CC BY 4.0 license.

São Paulo/SP and Natal/RN, which are hotspots for dengue transmission in the Latin American region [21]. In 2024 alone (up to September), São Paulo recorded more than two million cases of dengue fever and 1,600 deaths during this period [21]. In Natal, the capital of Rio Grande do Norte State, dengue reappeared in 1996. Since that time, the city has experienced recurring outbreaks, and in 2024, the number of dengue cases surged by 160% compared to the corresponding period in 2023 [21]. We collected meteorological information and dengue cases of both cities (Fig 1). These are two cities with very different climatic and socioeconomic conditions. The characteristics of each are described in Table 1. At approximately 12 million inhabitants, São Paulo is one of the most populous cities in the world [22]. São Paulo is Brazil’s major financial and economic center, contributing about 11.4% of total Brazilian gross domestic product [22].

**Table 1:** Climatic and demographic characteristics of the two cities analyzed: São Paulo/SP and Natal/RN. Data obtained from IBGE [22] and INMET [23].

Data	São Paulo/SP	Natal/RN
Population (inhabitants)	11,895,578	751,300
Area (km²)	1,580	167
Human density (hab/km²)	7,528	4,498
Climate	Humid subtropical	Humid coastal tropical
Seasons	Rainy summer – dry winter	Dry summer – mild/rainy winter
Average annual temperature (ºC)	20.0	26.0
Absolute Maximum temperature (ºC)	37.8	34.4
Absolute Minimum temperature (ºC)	3.5	18.2
Average Thermal amplitude (ºC)*	15.1	7.4
Average Total yearly rainfall (cm)*	218.8	392.5
Tree planting on public roads (%)	74.8	44.7
Ideal sewage system (%)	92.6	61.8

* Considering the period from 2014 to 2023.

Within the city of Natal, additional arboviruses, including Zika virus and chikungunya, were simultaneously present, with initial occurrences documented in 2015. Regarding these infections, during the period from 2015 to 2016, Natal-RN accounted for over half of the recorded morbidity burden in Rio Grande do Norte and approximately one-third of verified microcephaly cases linked to Zika virus transmission.

We gathered climatic data from weather stations in the municipalities of São Paulo and Natal, Brazil [23], covering the period from 2014 to 2023. Daily meteorological readings from each station were aggregated by week, and the arithmetic mean across all stations was calculated to produce overall weekly values for ambient temperature (mean, minimum, and maximum), rainfall, and the number of rainy days. Concerning heatwaves, we adhere to the definition provided by Seah et al. [24], who describe them in their research as “continuous intervals of elevated temperatures occurring within each epidemiological week”. We also investigated the effects of maximum temperature on dengue infections. We defined a heatwave as one when there were two or more days where the daily maximum temperature exceeded the 90th percentile of its historical distribution. We obtained weekly reports of confirmed dengue infections aggregated by onset date from 2014 and 2023 from the Brazilian Ministry of Health [25].

### Statistical analysis

#### Distributed lag non-linear model.

This approach builds upon the methodology of Seah et al. [24], who examined the connection between heatwaves and dengue fever in Singapore. Taking into account the typical mosquito lifespan (10–14 days), the human infectivity period (7 days) [26], the extrinsic incubation period in *Aedes* mosquitoes (8–12 days), and the intrinsic incubation period in humans (3–14 days) [27], the complete dengue transmission cycle lasts approximately 28–47 days, or about seven weeks. Based on this, we considered both the immediate effects at lag week 0 and the delayed effects of meteorological variations for lag weeks 1–6, covering a total lag period of seven weeks (weeks 0–6). This approach aligns with the lag duration reported by Sang et al. in a previous study [28].

Building on previous studies that identified non-linear relationships between temperature and dengue incidence [29,30], we employed the Distributed Lag Non-Linear Model (DLNM) package in R (version 2.3.9) [31] to examine the temporal effects of heatwaves on weekly reported dengue cases over the maximum lag period. The DLNM framework captures both non-linear and delayed associations between meteorological variables and dengue incidence by combining basis matrices for the exposure–response and lag–response relationships into a bi-dimensional cross-basis function, allowing these effects to be modeled simultaneously [31].

#### Negative Binomial Regression.

Our primary focus was on dengue case counts in São Paulo/SP and Natal/RN from 2014 to 2023. To analyze count data (positive integers), especially in cases of overdispersion – where the variance exceeds the mean – we employed negative binomial regression models. We first examined univariate associations between each variable and the outcome, then included all significant predictors in subsequent multivariable model iterations. Model significance was assessed using the omnibus test, while the significance of individual parameters was evaluated with the Wald Chi-Square test. Variables with p-values above 0.05 in the Wald Chi-Square test were excluded from the multivariable model. For models meeting all significance criteria, we compared Akaike Information Criterion (AIC) values, selecting the model with the lowest AIC as the best fit. Results from the binomial regression are expressed as incidence rate ratios (IRRs), calculated as the exponentiated beta coefficients (β). The IRR provides a relative measure for comparing incidences between two events [32].

## Results

**Fig 2:**
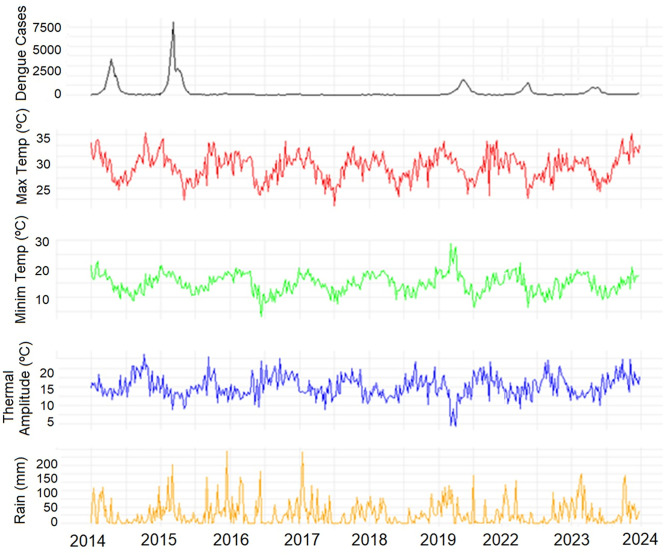
Time trend for weekly dengue infections, maximum and minimum temperature, thermal amplitude and rainfall in São Paulo/SP, Brazil, 2014–2023. *The years of 2020 and 2021 were excluded.

During the 417 epidemiological weeks of the study, a total of 149,468 dengue cases were reported in São Paulo city, while Natal recorded 80,999 cases. The years 2020 and 2021 were excluded from the analysis to mitigate potential underreporting caused by the COVID-19 pandemic. Moreover, a substantial amount of data was missing from conventional meteorological stations during this time frame. São Paulo averaged 335 dengue infections per week, with a high standard deviation of 896, indicating substantial weekly variability. The minimum number of infections was 0, and the maximum was 9,319 cases in a week, highlighting significant peaks of dengue during the period (Fig 2). Maximum and minimum temperatures remained fairly consistent throughout the weeks, exhibiting only moderate fluctuations. However, the thermal amplitude indicated a notable variation in daily temperatures during many weeks. Total precipitation showed great variability, with many dry weeks and some weeks with high rainfall volumes, which may be related to seasonal variability and its impact on the spread of dengue, considering the role of rainfall in the proliferation of the *Aedes aegypti* mosquito, the vector of the disease.

**Fig 3:**
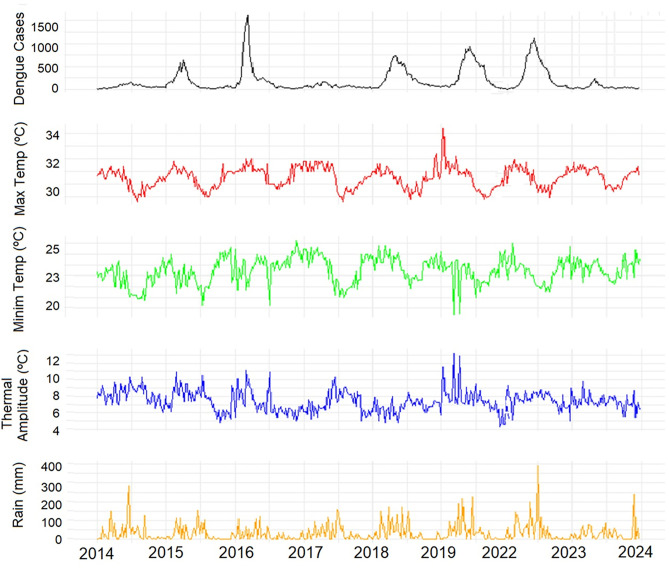
Time trend for dengue infections, maximum and minimum temperature, thermal amplitude and rainfall in Natal/RN, Brazil, 2014–2023. *The years of 2020 and 2021 were excluded.

In Natal, the weekly average of dengue infections was 189, with a standard deviation of 250, showcasing substantial variability in case numbers over time, though not as extreme as in Sao Paulo. Weekly maximum and minimum temperatures were constant, with small variations and generally warm temperatures, typical of a tropical climate (Fig 3). The temperature range in Natal was smaller than in Sao Paulo, averaging 7.4 °C, which is expected due to the city’s proximity to the sea and to the Equator, moderating daily temperature variations.

**Fig 4:**
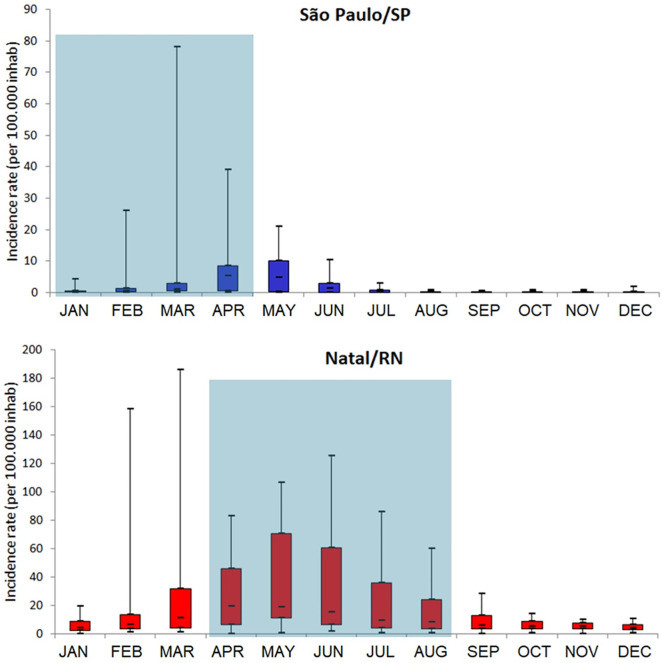
Seasonality of dengue infections in São Paulo and Natal, Brazil, 2014-2023. The blue box represents the rainy season. *The years of 2020 and 2021 were excluded.

Dengue infection peaks in Natal are more regular and recurrent than in São Paulo, with outbreaks typically occurring every two to three years. Natal’s maximum temperature shows a slight upward trend, while São Paulo’s shows a slight decrease. Minimum temperatures are relatively stable in both cities. Rainfall in Natal is concentrated in abrupt peaks, whereas São Paulo’s rainfall is more evenly distributed. Fig 4 demonstrates a strong seasonal pattern in dengue infections for both cities, with peaks coinciding with the rainy season.

We used a negative binomial model to analyze dengue infection counts in both cities. Model selection involved iteratively adjusting a full model, initially containing all independently and significantly associated variables (excluding average annual temperature and number of rainy days due to collinearity with minimum temperature and total rainfall, respectively), until a satisfactory predictive performance was achieved (Table 2). The final model significantly outperformed the null model (ANOVA, p < 0.0001), with a lower Akaike Information Criterion (AIC) (210.3 vs. 300.5). Minimum temperature showed a positive association with dengue infections, while maximum temperature and total rainfall were negatively associated.

**Table 2:** Test of model effects and parameter estimates for the final selected predictive model for dengue incidence in São Paulo/SP and Natal/RN cities, Brazil.

Variable	Estimate	Std. Error	*p* value	RR
Maximum temperature (ºC)	− 2.7 (- 4.3, – 1.1)	0.81	0.001	0.97
Minimum temperature (ºC)	1.8 (0.5, 4.2)	0.76	0.001	1.07
Thermal amplitude (ºC)	1.1 (- 1.3, 3.5)	2.98	0.356	1.03
Total rainfall (cm)	− 4.7 (- 9.7, 0.2)	2.56	0.005	0.95

**Fig 5:**
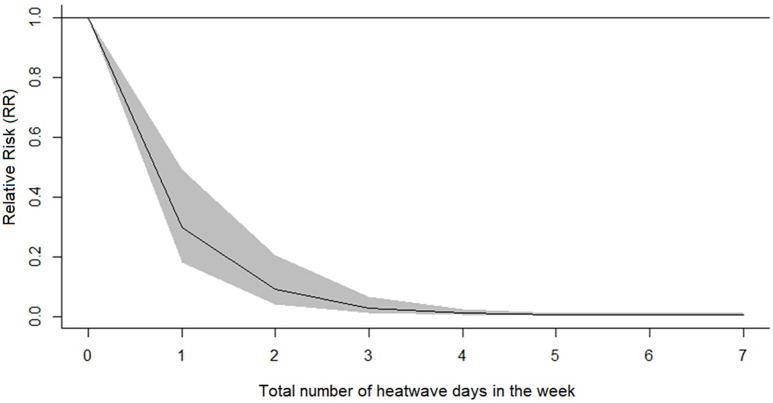
Exposure-response curve of São Paulo/SP, Brazil, showing overall cumulative effect of the total number of heatwave days in the week, with reference to no heatwave days in the week. Shaded areas represent 95% confidence intervals.

Fig 5 illustrates the relationship between heatwave days in a week and reported dengue infections in São Paulo. Natal experienced no heatwaves during the study period, so this analysis focuses solely on São Paulo, where most heatwaves lasted 1–2 days. We observed a reduced risk of dengue infection with increasing heatwave days (1–5) in a week. A single heatwave day was associated with a 70% reduction in risk (RR: 0.30, 95% CI: 0.18, 0.49). The risk continued to decrease with more heatwave days, reaching RR 0.09 after two days and 0.006 after five days. The risk reduction appears to plateau after three days, with minimal further decreases observed for 5–7 days. All relevant data for conducting this study are included in the Supplementary Material (S1 File).

## Discussion

This research offers new perspectives on how heatwaves and meteorological factors influence dengue infections in two Brazilian cities, adding to the expanding body of knowledge regarding the effects of global warming on arboviruses. A key finding of this study is the negative correlation between heatwaves and dengue cases. Heatwaves can influence dengue transmission in diverse ways, depending on their intensity, duration, and timing during the transmission season. Milder or short-term heatwaves may temporarily reduce mosquito activity or hinder viral replication due to thermal stress, which may help explain the decreased transmission observed in our findings. In contrast, longer or more intense heatwaves can produce more complex outcomes. From an environmental perspective, extended periods of high temperature can cause shallow water sources to evaporate, limiting mosquito breeding habitats. On a physiological level, extreme heat may induce a synchronized emergence of adult mosquitoes, followed by high mortality rates if the heat persists beyond their tolerance limits. Additionally, larvae exposed to very high temperatures (e.g., 39°C) show a reduced expression of heat shock protein genes, which are vital for surviving thermal stress – suggesting a potential vulnerability under extreme conditions [33]. These multifaceted effects indicate that the relationship between heatwaves and dengue is not straightforward and may vary by region. Therefore, we emphasize the need for localized studies to better define and understand heatwave events in different contexts, especially as mosquito populations may adapt over time to changing climate conditions [15,34].

Temperature’s effect on dengue transmission occurs through various complex mechanisms [35–38], including its impact on the female mosquito’s reproductive cycle, feeding behavior, and the virus’s incubation period in both mosquitoes and humans [39,40]. A study examining how temperature influences the virus’s incubation period revealed that as the temperature increased from 25ºC to 30ºC, the incubation period shortened from an average of 15 days to 6.5 days [27]. Other studies have further clarified this relationship; for example, Damtew et al. [6] reported a 13% increase in dengue risk for every 1°C rise in high temperatures above baseline values. Optimal temperature ranges for dengue transmission have been identified as 14.8°C for minimum temperature [37] and 32–33°C for maximum temperature [36]. The temperature ranges used in the referenced studies correspond to those influencing the biology of the *Aedes* mosquito and dengue virus, which likely explains the observed relationship between temperature and dengue risk in the present study. Viral replication reaches its peak at around 35ºC, a temperature higher than the threshold at which mosquito survival and feeding behaviors start to diminish [41], thereby limiting dengue transmission. This could account for the higher relative risk linked to minimum and average temperatures compared to maximum temperatures [8].

In Natal, peaks of infections are more regular and recurrent than in São Paulo. However, the annual patterns of dengue cases do not necessarily indicate that each year exhibits the same level of incidence. For example, in 2017, dengue incidence was notably lower compared to other years. This could be attributed to various factors, such as the presence of a population susceptible to the predominant circulating serotype [42], given that dengue is caused by four distinct virus serotypes. Additionally, the intricate interplay between environmental factors and the four dengue serotypes might have played a role [43]. Furthermore, the reported number of dengue cases in 2017 was below expectations for both Brazil and Colombia [44], which could be linked to the prior Zika virus infections in these populations [45].

We observed a negative correlation between rainfall and dengue incidence. Although water is crucial for *Ae. aegypti* development in outdoor containers, heavy rainfall can wash away these breeding sites, thereby reducing mosquito populations [46]. Between 2014 and 2015, São Paulo experienced its most severe drought since 1930 [47]. Interestingly, confirmed dengue cases drastically increased during this period (Fig 2), despite precipitation levels being below average. Typically, the seasonal increase in dengue cases coincides with the rainy season, which occurs in São Paulo during the first half of the year (Fig 4). Similar patterns have been documented in the Magdalena River watershed in Colombia [48–50], where higher dengue incidence is also observed during the warmer and wetter months. These authors suggest temperature as the primary driver, as human-filled water containers decouple mosquito reproduction from precipitation, implying an association without causation. The connection between temperature and dengue incidence has been observed in various other studies [51–53]. In São Paulo, the slightly higher-than-normal temperatures observed during the 2014 and 2015 dengue outbreaks are particularly relevant given the concurrent El Niño conditions. These conditions persisted from late 2014 through all of 2015 [54,55], potentially exacerbating the temperature’s influence on dengue transmission.

Our model indicates that the risk of dengue infections decreases dramatically as the number of days with heatwaves increases. The extremely low RR values from three days onwards suggest that the impact of heatwaves stabilizes after a certain point, with hotter weeks consistently presenting a much-reduced risk of dengue infections. Similar patterns have been reported in Singapore [24], though research on the effects of heatwaves on vectors, including mosquitoes, remains scarce. A recent study from Hanoi, Vietnam, suggested that heatwaves could lead to larger dengue outbreaks in the following weeks [3]. Interestingly, the inhibitory impact of heatwaves on dengue infections observed in our study contrasts with these earlier findings. We attribute this discrepancy to potential differences in the climates of the cities being compared, and, importantly, to variations in the definitions used to identify heatwave events. Jia et al. [15] demonstrated that heatwaves generally suppress mosquito populations. Laboratory research has established that temperature plays a critical role in shaping important mosquito characteristics related to dengue transmission, such as fecundity, survival, development speed, and adult lifespan [56–58]. Although moderate temperature increases can promote mosquito growth, exposure to extreme heat significantly limits this development [15].

Furthermore, earlier studies have shown that exposure to extreme temperatures leads to the downregulation of multiple genes associated with heat shock proteins, which play a crucial role in protecting *Aedes* mosquitoes from heat stress [33,59]. The increased mortality risk during prolonged periods of extreme heat offers a biologically plausible explanation for the observed reduction in dengue transmission. This effect, coupled with potential changes in human behavior during heatwaves, may contribute to the negative association we found between dengue and extreme heat [3]. During periods of extreme heat, people tend to spend more time in air-conditioned spaces—which are widespread in São Paulo—thereby reducing contact between humans and mosquitoes and lowering the risk of dengue transmission [24].

Several studies in Brazil have examined the relationship between temperature and dengue incidence using a variety of modeling approaches. Distributed lag non-linear models (DLNM) have been applied, such as in São Luís, Maranhão, where temperature and rainfall were evaluated using negative binomial models, revealing significant associations between rainfall (with a 3-month lag) and dengue cases, although temperature effects were less pronounced [60]. Similarly, a long-term analysis in Rio de Janeiro indicated that epidemics were more likely when minimum temperatures exceeded 22 °C and summers were both hot and dry, highlighting temperature as a primary driver of seasonal transmission [61]. In addition, Alves et al. [62] applied a mechanistic SEI–SIR model in multiple Brazilian cities using satellite-derived temperature and rainfall inputs, capturing key features of the seasonal dengue cycle and the role of temperature-driven vertical transmission. These studies collectively underscore the importance of temperature – often in conjunction with rainfall – as a critical climatic factor influencing dengue outbreaks in Brazil. However, most have focused on average temperatures or lagged seasonal patterns rather than on the health effects of extreme heat events. Our study contributes to this literature by explicitly assessing the impact of heatwaves, a dimension largely overlooked in previous Brazilian models. This event-based approach adds important insights into how short-term climatic extremes may disrupt mosquito ecology and dengue transmission dynamics, complementing existing knowledge built on long-term temperature trends.

This study faced several limitations. First, dengue cases may have been underreported, as infections are not always documented at onset, resulting in a misalignment between the timing of exposure and case reporting. Second, our reliance on weekly data constrained our ability to accurately capture the initiation, duration, and frequency of heatwaves and their specific impacts on dengue incidence. Third, unmeasured factors – such as exposure assessment methods, land use patterns, and other environmental, social, and demographic variables – may have contributed to the observed variability. For instance, Schmidt et al. [63] found that infrastructural shortcomings (e.g., inadequate water supply) had a more pronounced effect on dengue transmission in Vietnam than climatic factors. Fourth, the lack of data prevented us from stratifying our analysis by dengue serotype, an area that future studies should address. Lastly, our analysis did not consider the potential influence of local dengue control programs, which could significantly affect annual infection rates. The effectiveness of interventions such as Natal’s VigiDengue program and São Paulo’s Municipal Dengue Control Program (PMCD) warrants further investigation and could explain some of the observed variations in dengue incidence [64,65].

## Conclusions

Our findings suggest that dengue incidence increases in association with rising minimum temperatures, whereas elevated maximum temperatures, the occurrence of heatwaves, and higher cumulative rainfall are associated with reduced transmission. These patterns underscore the need for an integrated, multisectoral approach to arboviral disease control – one that bridges human health, vector ecology, and environmental dynamics. Both São Paulo and Natal exhibited distinct patterns in dengue distribution. Notably, the effects of extreme heat events, particularly heatwaves, on dengue transmission remain poorly understood. Addressing this critical knowledge gap will be essential to inform adaptive public health strategies and ensure regionally tailored responses under a changing climate.

## Supporting information

S1 File Database with dengue cases and climate variables from the municipalities of São Paulo/SP and Natal/RN used in this study. (XLSX)
